# Effects of Income and Psychological Identification on the Mental Health of China’s Migrated Agricultural Population

**Published:** 2018-09

**Authors:** Jing XU, Yue TIAN, Siting WANG, Yixuan LU

**Affiliations:** College of Business, Yangzhou University, Yangzhou, China

**Keywords:** Psychological identification, China’s migrated agricultural population, Mental health

## Abstract

**Background:**

The Migrated Agricultural Population (MAP) of China continues to increase with the continuous development of urbanization. As MAP is a socially disadvantaged group, their mental health issues require urgent attention.

**Methods:**

An ordinary least squares regression model was established by using the newest survey data from the 2016 Chinese General Social Survey. Moreover, the effects of income and psychological identification on the mental health status of China’s MAP were also examined using the Stata 12.0 software. The differences in the examination results under the influence of gender, educational level, and marital factors were compared.

**Results:**

The mental health level of China’s MAP is affected by both income and psychological identification. Specifically, income has a more significant influence on men’s mental health, whereas psychological identification is more significant for women. The mental health of MAP with spouses or those who received secondary education also reflects the overall characteristics of the sample. By contrast, those without spouses or those who did not receive other forms of education are mainly affected by psychological identification. Additionally, the mental health of the unmarried group is mainly affected by the family’s actual income and subjective well-being based on the psychological identification.

**Conclusion:**

The influence of income and psychological identification on the mental health of China’s MAP shows population differences. Therefore, different emphasis should be placed on the interventions of mental health in various groups of MAP. This study can provide decision-making references for the mental health management and psychological pressure counseling of MAP.

## Introduction

To improve the quality of life, most of agricultural populations in Asia leave their homes to seek employment in cities, where the migration phenomenon is particularly noticeable (1). China’s rural-urban migration is the world’s largest, most influential, most difficult, and most problematic issue. As a positive effect of urbanization, these migrant workers have made outstanding contributions to economic development, but only few of them have the same status as the urban population. As these workers make up a socially disadvantaged group, their physical health problems have attracted widespread attention to scholars. However, little attention has been paid to the mental health problems of this group. In 2016, the suicide rate of the rural elderly population in China was 47/100,000 and three times that in the same period in the United States (2). In addition, a survey conducted by Chen revealed that among the 807 migrant workers assessed in Shenzhen, about 58.5% had depression, 17% had anxiety, and 4.6% had thought about suicide. Many among those surveyed belong to the migrated agricultural population (MAP), and even a small number of the agricultural population holding urban household registration still faces serious social integration barriers (3). Amid such grim social realities, the factors that affect the psychological health of China’s MAP are worth considering.

Generally, the factors that affect individual’s mental health are rather complex, and they are mainly related to heredity, external environmental stimuli, mental quality levels and psychological health factors. It is difficult to regulate genetics and mental quality levels which are endogenous factors, so studies on mental health in the field of social psychology are mainly based on the exploration of exogenous factors, and interventions are provided from the perspective of mental health care. As MAP is a newly emerging group in China’s social transformation, few special investigations have been made on the factors affecting their mental health. Relevant studies predominantly focus on peasant workers’ groups, including the effects of psychological pressure (4), cultural adaptation (5) and socio-economic status (6) on the psychological health. Income and psychological identification affect strongly the psychological pressure, cultural adaptation and socioeconomic status of peasant workers, which has also been confirmed by many scholars. However, does income and psychological identification have the same effect on the mental health of MAP?

### State of the Art Definition of China’s MAP

The concept of MAP first appeared officially in the “Twelfth Five-Year Plan” promulgated by the Chinese government in 2010. Once the concept was put forward, it received widespread attention from the whole society, while there was no consensus regarding the definition of MAP in academia. According to some scholars, MAP usually was a group of “drifting peasants” without residence registration (7). Other scholars regarded MAP as people who left rural areas for cities and engaged in non-agricultural production activities. Although a small number of MAP may have obtained urban residence registration because of work or land acquisition, most members of MAP have not (8). The inconsistent definition of MAP results from the lack of relevant statistical data and consideration of the research requirements. Despite of the variations in definitions, an increasingly common view is that MAP does not necessarily consist of peasant workers, and MAP includes the population living in a town with rural household registration. Considering that the research topic of this article is related to mental health, for a specific and targeted study, MAP should be defined as the rural registered population that is or has ever been engaged in non-agricultural production activities and peasants who have obtained urban household registration because of non-subjective willingness factors, such as land acquisition and house demolition.

### Mental health of MAP

Mental health is an important part of health and it’s also a hot issue (9). Generally, mental health includes the emotional, psychological and social well-being of individuals. It varies from different perspectives. There are few studies on the mental health of MAP from western scholar, because MAP is a special product of social transformation and development under the influence of China’s household registration system, which is absent in western countries. Related studies focused mainly on farmers’ suicide issues (10), and extended investigations were conducted on farmers’ psychological health problems (11). Among the research of Chinese scholars on the mental health issues of MAP, the most measured mental health levels belonged to those of peasant workers, followed by those of landless peasants. The Symptom Check List 90 was used in the majority of investigations. Through their measurements, some academics considered the level of mental health of peasant workers as higher than that of ordinary people (12), whereas others believed that the level was significantly lower than those of normal adults (13). In general, the mental health level of peasant workers in China improved from 1995 to 2011. However, the mental health level of young peasant workers was declining, especially in terms of hostility and anxiety (14).

### Influence of income and psychological identification on the mental health of MAP

Some scholars emphasized that the social and economic status of the family influenced individual’s mental health, and it was more susceptible to chronic physical diseases and serious psychological problems for the low and middle-income families (15). To a large extent, the resources and services needed to maintain individual mental health and guarantee the future development of family members were inseparable from the family’s economic foundation. Moreover, the worse the social and economic status, the greater the mental pressure (16). In a study on the first-generation Latino adults’ depressive symptoms and well-being, Roy et al. found that the outcome variable was adjusted by the predictor variable of household income and neighborhood income (17). Also, Musonda confirmed that the income level played a significant role in regulating the results in the study of the effects of domestic violence, mental health and well-being (18). Obviously, these scholars have affirmed the effect of income on individual mental health. However, according to the analysis above, MAP is identical and specific to the general population. How income (8) affects the mental health of MAP remains unexplored. Accordingly, the following hypotheses have been proposed.

*H*_1a_: Actual individual income *I*_1_ has a significant effect on the mental health of MAP.
*H*_1b_: Actual family income *I*_2_ has a significant effect on the mental health of MAP.
*H*_1c_: Self-evaluation of personal income *I*_3_ has a significant effect on the mental health of MAP.
*H*_1d_: Self-evaluation of family income *I*_4_ has a significant effect on the mental health of MAP.

The effect of psychological identification on the individual mental health has also been studied in this research. From existing articles, western scholars largely focused on gender identification (19), social identification (20), cultural identification (21), and racial identification (22). The research objects included women (23), international students (21) and general adults (24). The results show that identification exerts an important influence on the mental health, and high continuity of social identification often indicates high life satisfaction and low depression level (25). The concrete representations of psychological identification vary from different groups. Hence, the psychological identification of MAP in the study mainly refers to a kind of psychological perception and evaluation of MAP while living and working around urban and rural areas. Because of the duality of the identity of MAP, they are neither traditional peasants nor conventional citizens, and great mobility makes them face rural and urban identification. The inherent representation of identity identification confusion refers to the issue of psychological identification, and it also leads to further social conflicts. Due to the short time MAP, few studies have been conducted on the relationship between their psychological identification and mental health in the world. However, psychological identification undoubtedly affects the belonging sense and social integration of MAP, and then brings relative deprivation and strong loneliness, inferiority, and frustration, which are essential for individuals to maintain mental health (26). Therefore, in accordance with Zhang et al. (8), psychological identification of MAP in the study is measured from four aspects, namely, satisfaction with government work *P*_1_, subjective well-being *P*_2_, the degree of trust in social members *P*_3_ and understanding of the development opportunities for future generations *P*_4_. The following hypotheses are proposed.

*H*_2a_: *P*_2_ has a significant effect on the mental health of MAP.
*H*_2b_: *P*_3_ has a significant effect on the mental health of MAP.
*H*_2c_: *P*_1_ has a significant effect on the mental health of MAP.
*H*_2d_: *P*_4_ has a significant effect on the mental health of MAP.

## Methods

### Data resources

Data in this article came from the newest 2016 Chinese General Social Survey (CGSS, www.chinagss.org) announced at the end of 2017. The CGSS systematically and comprehensively collects data from multiple levels of society, communities, families, and individuals. The personal survey was conducted in 28 provinces, including 125 counties (districts), 500 neighborhoods (villages and towns), approximately 1000 home (villages) committees and more than 10,000 families. At present, data from the CGSS have become the most important source for the study of Chinese society. Regarding to the source of the scales in this article, the income and psychological identification scales were selected from Zhang et al. (8). The dependent variables of mental health *Y* were obtained from the direct questions of the CGSS.

### Data description

According to the above definition of MAP, the total sample, which chiefly included two parts, was screened. Firstly, the surveyed samples who were or had engaged in non-agricultural production were selected from the population with agricultural residence registrations. Secondly, the surveyed samples whose agricultural residence registrations changed to non-agricultural residence registrations were selected due to some reasons such as land acquisition (including village reform) or residence registration reform. Through the summation of the two parts, the questionnaires that were invalid or had obvious errors were excluded, and 2486 research samples were obtained. According to the overall characteristics of the samples, 1461 males and 1025 females were surveyed. The academic qualifications below the university level account for 91.87%, which accorded the current basic conditions in China. In terms of marital status, the proportion of first current marriages was 80.49% (Table 1). In general, the overall structure of the data is balanced, and the sampling is logical and applicable for analysis.

**Table 1:** Statistics of the sample

***The highest education level***	***Sex***		***Total***
	***Male***	***Female***	
Without any education	73	120	193
Private school or literacy classes	11	5	16
Primary school	347	267	614
Junior high school	624	397	1021
Vocational high School	23	11	34
Normal high school	197	91	288
Secondary school	58	42	100
Technical school	13	4	17
University College (Adult high education)	22	14	36
University College (Formal high education)	40	36	76
Undergraduate (Adult high education)	12	8	20
University undergraduate (Normal high education)	35	26	61
Postgraduate and above	3	2	5
Other	3	2	5
Total	1461	1025	2486

### Data statistics

The basic situation is shown in Table 2. As the continuous variables *I*_1_ and *I*_2_ are actual values, there will be large errors if they are used directly.

**Table 2:** Explanation of the index

***Index***	***Variable***	***Item***	***Variable description***	***Variable type***
Income	*I*_1_	What is your total individual income last year (2014)?	Actual value	*C*
	*I*_2_	What is your total household income last year (2014)?	Actual value	*C*
	*I*_3_	Compared with your peers, what do you think of your own socio-economic status?	Grade 1-3. 1. Relatively low 2. General 3. Relatively high	*D*
	*I*_4_	Which level is your family’s economic situation in the region	Grade 1–5. 1. Well below average 5. Well above average.	*D*
Psychological identity	*P*_1_	Are you satisfied with the performance of government departments’ handling matters impartially?	Grade 1–5. 1.Very satisfied. 5. Very dissatisfied	*D*
	*P*_2_	In general, do you think your life is happy or not?	Grade 1–5. 1. Very unhappy. 5. Very happy	*D*
	*P*_3_	Do you agree that the majority of people in this society can be trusted?	Grade 1–5. 1. Strongly disagree. 5. Strongly agree	*D*
	*P*_4_	In our society, the descendants of workers and farmers have as many opportunities to become wealthy and powerful people as other people’s offspring.	Grade 1–5. 1. Strongly agree. 5. Strongly disagree	*D*
Mental health	*Y*	What is the frequency that you felt depressed or gloomy in the past 4 weeks?	Grade 1–5. 1. Always 5. Never	*D*

Note: *C*- the continuous variable, *D*-the virtual qualitative variable

Therefore, they were processed in logarithmic form. Then the reliability of the scale was analyzed, and the Cronbach’s α reliability coefficient of income, psychological identification, and overall scale was found to be 0.708, 0.5882, and 0.5834, respectively, which were all greater than 0.5. Therefore, the sample data were considered able to reflect the real situation of MAP, and follow-up analysis could be conducted.

### Model building

Based on the above data, a multiple regression models was established (see Model 1). The random variable *ɛ*, where *α* is a constant term, was added to avoid the influence of random error terms.

(1) $$Y=\alpha +\beta_{1}\text{ln}I_{1}+\beta_{2}\text{ln}I_{2}+\beta_{3}I_{3}+\beta_{4}I_{4}+\beta_{5}P_{1}+\beta_{6}P_{2}+\beta_{7}P_{3}+\beta_{8}P_{4}+ɛ$$

For enhanced analysis and comparison, the impact of income on the mental health of MAP (see Model 2) was studied. Then, the influence of psychological identification (see Model 3) was explored.

(2) $$Y_{1}=\alpha_{1}+\beta_{11}\text{ln}I_{1}+\beta_{12}\text{ln}I_{2}+\beta_{13}I_{3}+\beta_{14}I_{4}+{ɛ}_{1}$$

(3) $$Y_{2}=\alpha_{2}+\beta_{21}P_{1}+\beta_{22}P_{2}+\beta_{23}P_{3}+\beta_{24}P_{4}+{ɛ}_{2}$$

## Results

According to the above hypothetical model, the regression analysis was carried out, as shown in Table 3. Although the regression results are significant, *R*^2^ in all three models are not large, indicating that the overall explanatory power is weak.

**Table 3:** Regression results of mental health of MAP affected by income and psychological identification

***Variable***	***Model 2***		***Model 3***		***Model 1***	
	***Coef.***	***T***	***Coef.***	***T***	***Coef.***	***T***
*lnI*_1_	0.04	1.56			0.05^*^	1.73
*lnI*_2_	0.09^***^	3.12			0.07^**^	2.39
*I*_3_	0.21^***^	4.72			0.14^***^	3.24
*I*_4_	0.07^**^	1.88			0.01	0.22
*P*_1_			0.05^***^	2.98	0.04^**^	2.26
*P*_2_			0.36^***^	13.89	0.31^***^	11.25
*P*_3_			0.01	0.33	0.01	0.27
*P*_4_			−0.02	−0.86	0.01	0.37
Constant	2.77^***^	9.73	2.34^***^	17.66	1.67^***^	5.47
R^2^	4.62%		7.59%		9.60%	
Adj *R*^2^	4.47%		7.44%		9.30%	
Prob > *F*	0.0000		0.0000		0.0000	

Note:

* indicates *P*<0.1;

** indicates *P*<0.05;

*** indicates *P<*0.01

However, given the various factors affecting mental health, income and psychological identification constitute only a small portion of such factors, and there are many samples, so the results of this work can be used as an explanatory analysis for the research into non-predictive social issues. Overall, income can explain 4.62% of the mental health status of MAP, and psychological identification 7.59%, and they together 9.58%. Specifically, only considering the effect of income on the mental health of MAP, the influence of *I*_1_ is not significant (*p*=0.118), while *I*_2_ (*T*=3.12) and I_3_ (*T*=4.72) exert significant effects. If only considering the effect of psychological identification, the influence of *P*_2_ is obviously most significant (*T*=13.89), followed by *P*_1_ (*T*=2.98). However, *P*_4_ (*T*=0.37) and *P*_3_ (*T*=0.27) have no significant effect on the mental health. When considering the influence of income and psychological identification simultaneously, the effect of *I*_1_ on the mental health changes from insignificant to significant (*T*=1.73), while the effect of *I*_4_ changes from significant to insignificant (*T=*0.22). It indicates that, if considering subjective well-being, the absolute value of actual individual income has a stronger influence on the mental health of MAP than the relative household income.

The internal differences in the effects of income and psychological identification on the mental health of MAP were further explored. Specifically, the influence of gender, educational level, and marital status was also analyzed. Considering that *P*_3_ and *P*_4_ have no significant influence on the mental health of MAP for Model 3 or Model 1, they were eliminated in subsequent analyses.

### Influence of gender differences

The least squares method for regression analysis was further applied to investigate the mental health of MAP influenced by gender differences. *I*_1_ has no significant effect on *Y* of males (*T*=1.55) and females (*T*=0.35), while *I*_2_ (*T*=1.99) and *I*_3_ (*T*=2.94) have a significant effect on the mental health of males but not on that of females. On the contrary, *I*_4_ has no significant effect on the mental health of males (*T*=−1.47), but the impact on *Y* of females is significant (*T*=2.21). For the psychological identification index, *P*_1_ has no significant effect on the mental health of males (*T*=−0.23) but has an extremely significant impact on that of females (*T*=3.22). In general, the higher the income and psychological identification level, the lower the frequency of depression, which can be seen in Table 4.

**Table 4:** Effects of gender differences on the mental health of MAP

***Variable***	***Male***		***Female***	
	**Coef.**	***T***	**Coef.**	***T***
*lnI*_1_	0.06	1.55	0.01	0.35
*lnI*_2_	0.08^**^	1.99	0.07	1.58
*I*_3_	0.17^***^	2.94	0.10	1.51
*I*_4_	−0.07	−1.47	0.12^**^	2.21
*P*_1_	−0.01	−0.23	0.07^***^	3.22
*P*_2_	0.28^***^	7.89	0.33^***^	8.31
Constant	1.94^***^	5.16	1.41^***^	3.06
R^2^	8.38%		12.29%	
Adj *R*^2^	8.01%		11.78%	
Prob > *F*	0.0000		0.0000	

Note:

* indicates *P*<0.1;

** indicates *P*<0.05;

*** indicates *P*<0.01

### Influence of educational levels

The influence of educational level differences on the mental health of MAP was studied, and the results were shown in Table 5. *I*_2_ (*T*=2.18), *I*_3_ (*T*=3.27), *P*_1_ (*T*=2.17) and *P*_2_ (*T*=9.61) exert a significant effect on the mental health of MAP whose educational level is from primary school to high school (Edu.2). For those whose educational level is below primary school (Edu.1) and university or above (Edu.3), the effect of income on *Y* is not significant (*P*>0.1), and only *P*_4_ (*T*=5.27, 4.65) has an obvious effect. The influence of individual income on *Y* in Edu.3 is not significant but shows a certain negative correlation (Coef.= −0.04), indicating that the higher their individual income is, the higher the psychological pressure they can be under.

**Table 5:** Effects of educational differences on the mental health of MAP

***Variable***	***Edu.1***		***Edu.2***		***Edu.3***	
	**Coef.**	***T***	**Coef.**	***T***	**Coef.**	***T***
*lnI*_1_	0.01	0.08	0.05	1.51	−0.04	−0.69
*lnI*_2_	−0.04	−0.57	0.08^**^	2.18	0.02	0.21
*I*_3_	0.13	1.05	0.16^***^	3.27	0.01	0.07
*I*_4_	0.06	0.54	−0.002	−0.05	0.04	0.39
*P*_1_	0.03	0.58	0.04^**^	2.17	−0.07	−1.13
*P*_2_	0.39^***^	5.27	0.29^***^	9.61	0.36^***^	4.65
Constant	2.53^***^	3.30	1.41^***^	3.06	3.02^***^	3.29
R^2^	16.96%		8.52%		14.03%	
Adj *R*^2^	14.50%		8.25%		11.33%	
Prob > *F*	0.0000		0.0000		0.0001	

Note:

* indicates *P*<0.1;

** indicates *P*<0.05;

*** indicates *P*<0.01.

### Influence of marital status

The sample was divided into three groups according to their marital status: unmarried, married (including cohabitation, first marriage, and remarriage) and divorced or widowed groups. The sample numbers are as follow: unmarried of 243, married of 2076 and divorced or widowed of 167, and the regression results are shown in Table 6. For singles in MAP, the main factors affecting *Y* include *I*_2_ (*T*=2.27) and *P*_2_ (*T*=3.91). For married individuals, besides psychological identification factor, a major effect on *Y* is individual income, including *I*_1_ (*T*=1.97) and *I*_3_ (*T*=2.91), while the effect of household income is not obvious (*P*>0.1). For divorced or widowed people, only *P*_2_ (*T*=3.51) has a significant effect. In addition, considering that most unmarried people are living with their parents and did not engage in agricultural production activities, their income accounts for a large portion of the total household income. As a result, the group with high individual income is highly likely to suffer from mental stress (Coef.= −0.03).

**Table 6:** Effects of marriage differences on the mental health of MAP

***Variable***	***Unmarried***		***Married***		***Divorced or widowed***	
	**Coef.**	***T***	**Coef.**	***T***	**Coef.**	***T***
*lnI*_1_	−0.03	−0.41	0.06^**^	1.97	0.06	0.65
*lnI*_2_	0.18^**^	2.27	0.04	1.28	0.06	0.60
*I*_3_	0.14	1.12	0.14^***^	2.91	0.18	1.12
*I*_4_	−0.08	−0.79	0.02	0.06	−0.03	−0.21
*P*_1_	−0.03	−0.43	0.04^*^	1.91	0.06	1.22
*P*_2_	0.29^***^	3.91	0.30^***^	10.2	0.34^***^	3.51
Constant	1.83^**^	2.23	1.81^***^	5.54	1.60	1.37
R^2^	12.46%		8.81%		13.74%	
Adj *R*^2^	10.23%		8.55%		10.51%	
Prob> *F*	0.0000		0.0000		0.0005	
Obs	243		2076		167	

Note:

* indicates *P*<0.1;

** indicates *P*<0.05;

*** indicates *P*<0.01

## Discussion

Firstly, individual and household incomes affect the mental health (Hypotheses *H*_1a_–*H*_1d_ are tenable), but the effects extent is also influenced by demographics. Secondly, subjective well-being and satisfaction with the fairness of the government’s law enforcement exert a significant effect on the mental health (Hypotheses *H*_2a_ and *H*_2c_ are tenable).

By contrast, awareness of the opportunities for the development of future descendants and the degree of trust in social members are not significant (Hypotheses *H*_2b_ and *H*_2d_ are not tenable). Generally, for MAP, high income and strong psychological identification equate to low possibility of mental depression. However, restricted by the material conditions, more attention has been paid to the subjective feelings in real life, showing a relative shortsightedness in view of the factors that affect their mental health. Therefore, the awareness of the opportunities for the development of future descendants do not have a significant effect on their mental health, while the income level, individual well-being, and satisfaction with the fairness of the government’s law enforcement can significantly affect their mental health. According to the Maslow’s hierarchy of needs theory, China’s MAP is remaining at a low level of demand. Therefore, from the perspective of improving the mental health and reducing the depression, the government should firstly focus on enhancing their experience of material life.

Given the thought that “men go outside, and women stay” in Chinese traditional values, income has a significant effect on men’s mental health, whereas psychological identification substantially influences women’s mental health. Especially for MAP with a generally low educational level, earning money is the main responsibility of men, and maintaining the harmony and happiness of a family is the main obligation of women.

For the MAP who have not attended school and received a high education, only the sense of well-being has a significant effect on their mental health, while the other variables have less effect. However, MAP who receive primary school to high school education show the full characteristics of the sample, and their mental health is influenced by psychological identification and income. That’s because, for the MAP who have not been to primary school, the income expressed by the psychological expectations is consistent with or even higher than their actual income, so the possibility of feeling depressed on the income is quite low.

For the unmarried group, their mental health is mainly affected by the actual household income and the sense of well-being. The former maintains their material level, while the latter meets their spiritual needs. For the divorced or widowed group, their mental health is mainly influenced by the sense of well-being. For the most of the members of the MAP with spouses, their mental health is affected by income and psychological identification. Obviously, marital differences in the sample also represent their living status to some extent. Most people with spouses have the responsibility to “play roles in connecting the elderly and kids”, and their mental health can be significantly influenced by bad external stimuli.

## Conclusion

Aiming at the mental health of China’s MAP, the impact mechanism of income and psychological identification on the mental health was studied deeply using data from the newest 2016 CGSS. The sample expressions of the gender, educational level and marital status in this impact relationships were also analyzed. The empirical results show that individual income, household income, well-being and satisfaction about the government’s fair law enforcement significantly affect the mental health of China’s MAP. However, population differences can also be reflected, indicating that mental health counseling for various groups should grasp different directions. Therefore, the group differences in the mental health problems of China’s MAP should be considered, which can provide important practical significance in improving the psychological health care for China’s MAP.

## Ethical considerations

Ethical issues (Including plagiarism, Informed Consent, misconduct, data fabrication and/or falsification, double publication and/or submission, redundancy, etc.) have been completely observed by the authors.
